# Influence of *CYP2D6*, *CYP2C19*, and *CYP2C9* Pharmacogenetics and Clinical Factors on Dose-Normalized Venlafaxine/O-Desmethylvenlafaxine Metabolic Ratio in Spanish Patients

**DOI:** 10.3390/ph19020209

**Published:** 2026-01-26

**Authors:** Levin Thomas, Carla González de la Cruz, Carmen Mata-Martín, Idian González-Rodríguez, Idilio González-Martínez, Eva M. Peñas-Lledó, Adrián LLerena

**Affiliations:** 1 Personalised Medicine and Mental Health Unit, University Institute for Biosanitary Research of Extremadura (INUBE), 06080 Badajoz, Spain; levinpharma@gmail.com (L.T.); carla.gonzalezd@externos.salud-juntaex.es (C.G.d.l.C.); mariadelcarmen.mata@salud-juntaex.es (C.M.-M.); idian.gonzalez@salud-juntaex.es (I.G.-R.); idilio.gonzalez@salud-juntaex.es (I.G.-M.); 2 Clinical Pharmacology Service, Pharmacogenomics and Personalized Medicine Unit, Badajoz University Hospital, Extremadura Health Service (SES), 06006 Badajoz, Spain; 3 Psychiatry Unit, San Pedro de Alcántara Hospital, Extremadura Health Service (SES), 10002 Cáceres, Spain; 4 Psychiatry Unit, Llerena Hospital, Extremadura Health Service (SES), 06900 Llerena, Spain

**Keywords:** venlafaxine, O-desmethylvenlafaxine, CYP2D6, CYP2C19, pharmacogenetic

## Abstract

**Background/Objectives**: Venlafaxine has been reported to exhibit significant interindividual pharmacokinetic heterogeneity across populations, which has been linked to cytochrome P450 polymorphisms and clinical factors. This study aimed to assess the impact of pharmacogenetic (PGx) and clinical determinants on the dose-normalized venlafaxine/O-desmethylvenlafaxine (ODV) metabolic ratios (MRs) in routine clinical settings in Spain. **Methods**: 29 adult patients receiving venlafaxine were prospectively recruited through the MedeA PGx Implementation Strategy into clinical practice (Extremadura, Spain). *CYP2D6*, *CYP2C19*, and *CYP2C9* genotypes were determined using TaqMan^®^ assays, and *CYP2D6* activity scores were assigned based on allele functionality. Steady-state trough plasma concentration of venlafaxine and ODV were measured using a validated high-performance liquid chromatography method. Dose-normalized venlafaxine/ODV MRs were compared across *CYP2D6*-, *CYP2C19*-, and *CYP2C9*-genotype-predicted metabolizer groups. The influence of demographic and clinical variables on dose-normalized venlafaxine/ODV MR was also assessed. **Results**: Significant variability in dose-normalized venlafaxine/ODV MRs was observed across *CYP2D6* (*p* = 0.019) and *CYP2C19* (*p* = 0.008) metabolizer groups. Among clinical variables, sex was significantly associated with differences in dose-normalized venlafaxine/ODV MR (*p* = 0.0006). **Conclusions**: *CYP2D6* and *CYP2C19* genotypes and sex significantly contribute to variability in venlafaxine metabolism in patients treated in routine clinical settings. These results highlight the value of combining PGx and clinical data with drug plasma concentration measurement to optimize venlafaxine therapy within PGx implementation programs.

## 1. Introduction

Depressive disorders are highly prevalent across Europe, with an estimated current prevalence of 6.38% (95%CI: 6.24–6.52), representing a leading cause of global disease burden [1]. Venlafaxine, a serotonin–norepinephrine reuptake inhibitor (SNRI), is widely prescribed for the treatment of major depressive disorder [2] and other anxiety-related disorders [3]. Evidence from meta-analysis showed that venlafaxine was associated with a greater response than selective serotonin reuptake inhibitors (SSRIs) and tricyclic antidepressants in the treatment of major depressive disorders. Venlafaxine was also more effective for treatment-resistant depression and in reducing relapse when given long-term after a major depressive episode [4]. A retrospective study of SSRI-unresponsive inpatients reported that switching to venlafaxine may offer greater benefit than switching to another SSRI [5]. Venlafaxine undergoes extensive first-pass hepatic metabolism in humans, with only 1–10% of the administered dose being excreted in urine in unchanged form [6]. Venlafaxine undergoes metabolism, primarily through CYP2D6-mediated O-demethylation to the pharmacologically active metabolite O-desmethylvenlafaxine (ODV). CYP2C19 and CYP2C9 enzymes have also been implicated in venlafaxine biotransformation, but to a relatively lower extent [6,7]. Both venlafaxine and ODV, with mean plasma half-lives of approximately 5 and 11 h, respectively, exhibit linear kinetics over the dose range of 75–450 mg/day [8,9]. Several studies have reported sub-optimal clinical outcomes and large inter-individual variability in plasma concentrations of both venlafaxine and ODV [10,11,12,13]. Further, venlafaxine has a narrow therapeutic range and is also associated with a wide spectrum of adverse drug reactions (ADRs) [14,15,16,17,18,19]. Notably, venlafaxine has been among the most frequently reported drugs in the Spanish Pharmacovigilance Database (FEDRA^®^) associated with self-directed violent behaviors, including suicidal ideation and attempts [18]. A recent pharmacovigilance analysis of the EudraVigilance database (2001–2024) comparing six commonly prescribed antidepressants (duloxetine, citalopram, escitalopram, fluoxetine, venlafaxine, and sertraline) identified venlafaxine as the antidepressant most frequently associated with psychiatric ADRs and completed suicides, highlighting its comparatively elevated risk profile [20]. This marked inter-individual and clinical response variability, coupled with venlafaxine’s narrow therapeutic index and broad range of ADRs, underscores the growing importance of therapeutic drug monitoring (TDM) and pharmacogenetic (PGx)-guided venlafaxine prescribing to optimize efficacy and minimize toxicity.

PGx variability in cytochrome P450 drug-metabolizing enzymes plays a key role in the pharmacokinetic variability of venlafaxine. Previous studies have reported that *CYP2D6* and *CYP2C19* polymorphisms and metabolizer status significantly influence venlafaxine plasma concentrations [21,22,23]. Higher *CYP2D6* and *CYP2C19* activity scores (AS) have been associated with lower venlafaxine concentrations [24]. The U.S. Food and Drug Administration’s (FDA) Table of Pharmacogenetic Associations identifies a *CYP2D6* gene–drug interaction for venlafaxine, suggesting that dose reductions may be considered in *CYP2D6* poor metabolizer individuals [25]. The Spanish Agency of Medicines and Medical Devices (AEMPS) and the European Medicines Agency (EMA) mention that *CYP2D6* metabolizers have higher plasma concentrations than rapid metabolizers and extensive metabolizers, respectively; however, as the total exposure of venlafaxine and ODV is similar between these groups, different venlafaxine dosing regimens for these groups are not recommended [26,27]. The Clinical Pharmacogenetics Implementation Consortium (CPIC) guideline recommends considering a clinically appropriate alternative antidepressant not predominantly metabolized by the CYP2D6 enzyme for patients who are *CYP2D6* poor metabolizers (classification of recommendation: optional) [28]. The venlafaxine/ODV metabolic ratio (MR) accurately predicted the *CYP2D6* poor metabolizer (gPM) phenotype [29]. Furthermore, *CYP2D6* gPM status has been implicated as a potential risk factor for ADRs to venlafaxine [30]. Besides PGxs, several demographic and clinical factors, such as age, sex, and smoking, have been reported to affect the venlafaxine and/or ODV concentration [31].

Despite limited evidence directly linking *CYP2D6* and *CYP2C19* genotypes to venlafaxine biotransformation, the combined impact of PGx and clinical factors on its pharmacokinetic profile remains insufficiently characterized in real-world clinical settings. Therefore, the present exploratory study aimed to evaluate the influence of *CYP2D6*, *CYP2C19*, and *CYP2C9* genetic polymorphisms, demographic and clinical factors, on dose-normalized venlafaxine/ODV MR in patients treated with venlafaxine under routine clinical conditions.

## 2. Results

### 2.1. Patient Demographics, Clinical Parameters, and PGx Data

The study cohort comprised 29 adult patients treated with venlafaxine. The study population was predominantly female (86.21%), and 37.93% of the patients were aged 65 years or older. Most of the patients had a body mass index (BMI) ≥ 25 kg/m^2^ (75.86%), and nearly half were current or former smokers (44.83%). With respect to PGx profiles, *CYP2D6* intermediate metabolizers (gIMs) and normal metabolizers (gNMs) were most prevalent (48.28% and 44.83%, respectively), and gPMs were 6.90%. For *CYP2C19*, the gNM subgroup constituted the largest subgroup (44.83%), followed by the rapid metabolizers (gRMs) (27.59%) and gIMs (20.69%), with gPMs and ultrarapid metabolizers (gUMs) each accounting for 3.45%. For *CYP2C9*, gNMs predominated (58.62%), followed by gIMs (34.48%) and gPMs (6.90%). Allele and genotype distributions for *CYP2D6*, *CYP2C19*, and *CYP2C9* are provided in Supplementary Tables S2–S7 (Supplementary File S1). Polypharmacy was present in approximately one-third of patients (34.48%), and hyper-polypharmacy in 24.14%. Exposure to CYP-inhibiting co-medications was limited and predominantly involved weak inhibitors. Specifically, weak CYP2C19 inhibitors were observed in 9 patients (31.03%), with one additional patient (3.45%) receiving a moderate CYP2C19 inhibitor. In contrast, exposure to weak CYP2C9 inhibitors was identified in only 2 patients (6.90%), and the use of weak CYP2D6 inhibitors was rare, occurring in a single patient (3.45%). No patients were on concomitant medications that were strong inhibitors of CYP2D6, CYP2C19, or CYP2C9.

Venlafaxine + ODV (active moiety) concentrations within the recommended therapeutic range (100–400 ng/mL) were observed in 9 patients (31.03%). Active moiety concentrations <100 ng/mL occurred in 1 patient (3.45%), >400 ng/mL in 19 patients (65.52%), and >800 ng/mL in 10 patients (34.48%). Daily venlafaxine doses ranged between 37.5 mg and 300 mg, the median (Q1, Q3) values of venlafaxine plasma concentration was 86.70 ng/mL (29.95, 201.3); the ODV plasma concentration, 514.1 ng/mL (237.1, 756.1); active moiety plasma concentration, 643.1 ng/mL (299.9, 974.3); venlafaxine/ODV MR, 0.13 (0.06, 0.36); and dose-normalized venlafaxine/ODV MR, 0.0007 (0.0005, 0.0018). A moderate positive correlation was observed between total daily venlafaxine dose and venlafaxine plasma concentration (r = 0.47, *p* = 0.009). The mean ODV plasma concentrations were ~3.6 times higher than those of venlafaxine (557.5 ng/mL vs. 151.3 ng/mL).

### 2.2. Effect of CYP2D6, CYP2C19, and CYP2C9 Genotype Predicted Metabolizer Groups on Dose-Normalized Venlafaxine/ODV MR

**Figure 1:**
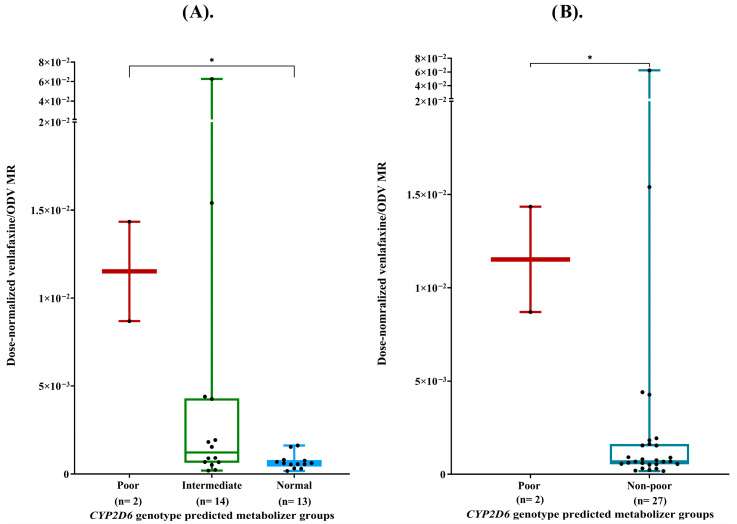
(**A**) Dose-normalized venlafaxine/ODV MR across different *CYP2D6* genotype predicted metabolizer groups. (**B**) Dose-normalized venlafaxine/ODV MR across *CYP2D6* poor and non-poor (gIMs + gNMs) metabolizers. Footnotes: * *p* < 0.05.

Significant differences in dose-normalized venlafaxine/ODV MR were observed across *CYP2D6* genotype predicted metabolizer groups (Figure 1A; *p*-value = 0.019). Post hoc pairwise analysis indicated that *CYP2D6* gPMs had a significantly greater MR than gNM (*p*-value = 0.045), while no other pairwise comparisons reached statistical significance (*p* > 0.05). Additionally, after grouping non-poor (gIMs + gNMs) metabolizers, *CYP2D6* gPMs exhibited a significantly higher dose-normalized venlafaxine/ODV MR compared to this combined non-poor group (*p* = 0.044) (Figure 1B).

**Figure 2:**
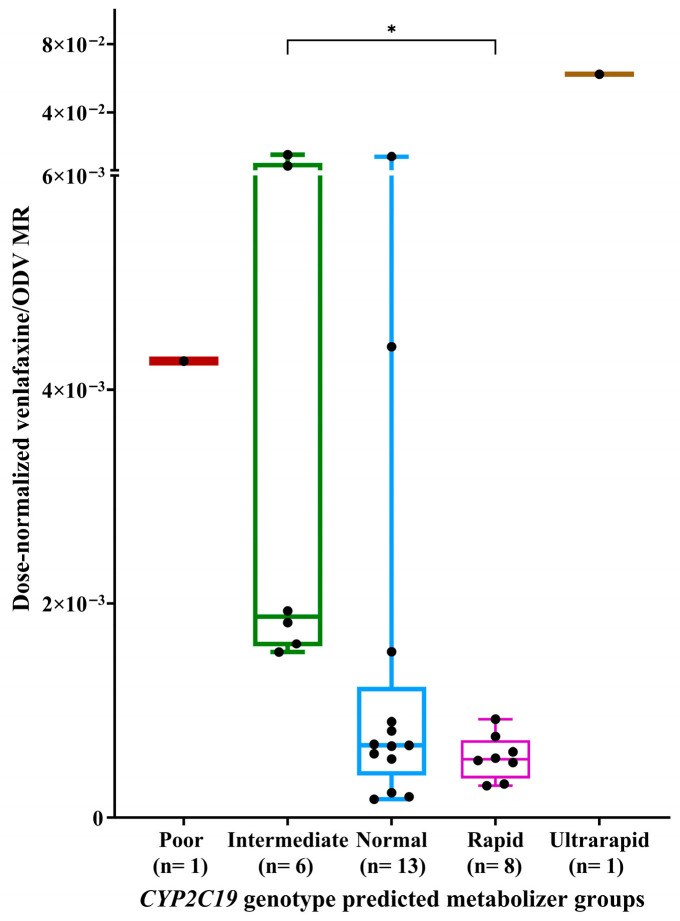
Comparison of dose-normalized venlafaxine/ODV MR among *CYP2C19* genotype predicted metabolizer groups. Footnotes: * *p* < 0.05.

Significant differences in the dose-normalized venlafaxine/ODV MR were observed among *CYP2C19* metabolizers (Figure 2; *p*-value = 0.008). Subsequent post hoc pairwise comparison indicated that patients classified as *CYP2C19* gIMs had significantly higher dose-normalized venlafaxine/ODV MR compared with gRMs (*p*-value = 0.027). No additional pairwise comparisons among *CYP2C19* metabolizer groups reached statistical significance (*p* > 0.05).

**Figure 3:**
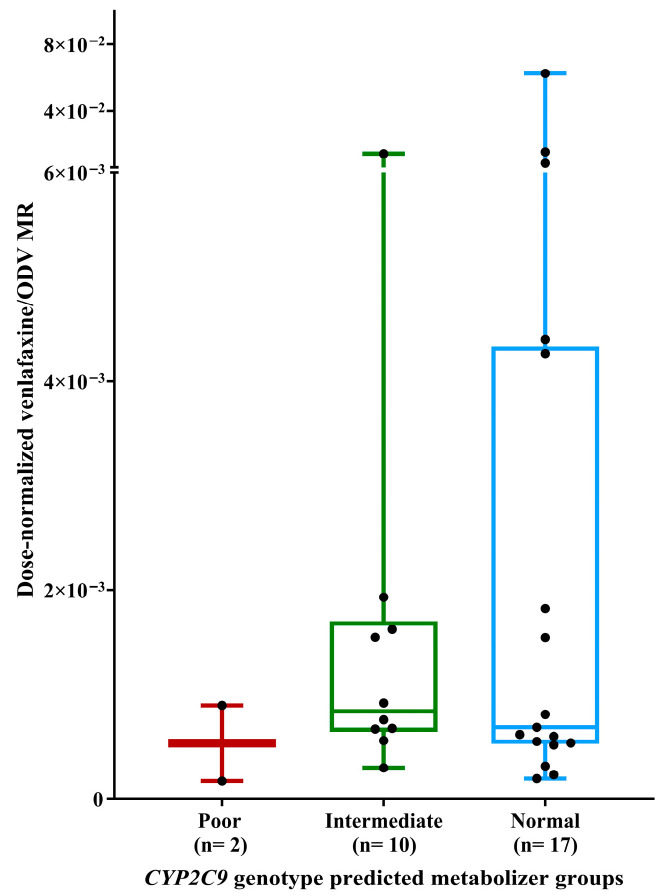
Comparison of dose-normalized venlafaxine/ODV MR across *CYP2C9* genotype predicted metabolizer groups.

No statistically significant differences were observed in the dose-normalized venlafaxine/ODV MR across CYP2C9 genotype predicted metabolizer groups (*p* = 0.551). Furthermore, post hoc pairwise comparisons confirmed the absence of statistically significant differences between any individual CYP2C9 metabolizer groups, as illustrated in Figure 3.

### 2.3. Impact of Demographic and Clinical Factors on Dose-Normalized Venlafaxine/ODV MR

Among the demographic and clinical variables analyzed, sex emerged as the only factor significantly associated with the dose-normalized venlafaxine/ODV MR (*p* = 0.0006), with males presenting considerably higher values compared to females (despite both having the same median total daily venlafaxine dose of 150 mg). No significant associations were observed between dose-normalized venlafaxine/ODV MR and older age, BMI, smoking status, comorbidity burden (≥5 comorbidities), diabetes mellitus, or hypertension (Table 1). The dose-normalized venlafaxine/ODV MR based on individual *CYP2D6*, *CYP2C9*, and *CYP2C19* single-nucleotide polymorphisms (SNPs) and total daily venlafaxine dose is shown in Supplementary Tables S8 and S9, respectively (Supplementary File S1). No individual *CYP2D6*, *CYP2C9*, or *CYP2C19* SNPs were significantly associated with dose-normalized venlafaxine/ODV MRs.

No statistically significant differences in dose-normalized venlafaxine/ODV MR were observed between patients with and without polypharmacy/hyperpolypharmacy status (*p* > 0.05). Intake of weak CYP2D6 and CYP2C9, and weak/moderate CYP2C19 inhibitors did not cause any increase in dose-normalized venlafaxine/ODV MR, as shown in Table 2.

## 3. Discussion

This study provides an initial assessment of the influence of *CYP2D6*, *CYP2C19*, and *CYP2C9* PGx and clinical factors on venlafaxine biotransformation, generating preliminary translational evidence to support psychiatric PGx-TDM implementation initiatives. *CYP2D6* and *CYP2C19* genotypes have been reported to influence venlafaxine plasma concentration in several ethnic populations, including Asian [23], Northern European [22], North American [24]. Marked population-specific gradients in *CYP2D6* and *CYP2C19* allele frequencies have been consistently reported across diverse ethnic groups, with substantial heterogeneity also observed among European countries [32,33]. These differences may influence the magnitude and clinical relevance of PGx. The present study adds value to the existing literature by providing the first real-world evidence from a Spanish population evaluating the influence of PGx, clinical characteristics, and polypharmacy-related factors, including DDIs, on dose-normalized venlafaxine/ODV MRs. In addition to the evaluation of *CYP2D6*, *CYP2C19*, and *CYP2C9* genotype predicted phenotype on the dose-normalized venlafaxine/O-desmethylvenlafaxine MR, we have also examined the independent effects of individual SNPs.

A recent systematic review and meta-analysis proposed target TDM ranges of 85– 380 ng/mL for ODV and 140–600 ng/mL for the active moiety for the treatment of depressive disorders [19]. In contrast, the recommended therapeutic reference range for the active moiety is 100–400 ng/mL, with concentrations exceeding 800 ng/mL considered a laboratory alert level according to the guidelines of the TDM task force of the “Arbeitsgemeinschaft für Neuropsychopharmakologie und Pharmakopsychiatrie” (AGNP) [34]. A moderate positive correlation was observed between total daily venlafaxine dose and venlafaxine plasma concentrations, indicating that systemic exposure increases with dose escalation. Notably, approximately one-third of the patients in this study had laboratory alert levels of the active moiety. These findings underscore the need for further research to assess the risk and incidence of ADRs. Elevated active moiety levels in certain patients may be attributable to factors such as advanced age, polypharmacy, and unaccounted drug–drug interactions. The mean ODV concentration was ~3.6 times higher than the mean venlafaxine concentration in our study. Reports from the Japanese population showed that ODV plasma concentrations were ~3.2 times higher than those of venlafaxine [35]. An open-label, fixed-dose study of venlafaxine (300 mg/day) among 35 depressed in-patients showed that patients who had an early response had a significantly higher active moiety concentration than those with a delayed response (median = 725 ng/mL vs. 554 ng/mL, *p* = 0.023) [36]. However, in a study of 52 elderly depressed outpatients in Italy, serum concentrations of the active moiety showed a bell-shaped relationship with antidepressant response; the response increased steadily between 100 and 400 ng/mL, peaked at around 400 ng/mL, and then declined at higher concentrations [12].

CYP2D6 is the primary enzyme involved in the biotransformation of antidepressants such as venlafaxine and fluoxetine [21,30,37]. Consistent with present findings, population pharmacokinetic [38], physiologically based pharmacokinetic [39] modeling, and TDM analyses [29] have shown that patients with *CYP2D6* gPM status exhibit significantly reduced venlafaxine metabolism, resulting in elevated venlafaxine exposure and consequently higher dose-normalized venlafaxine/ODV MR. The observed influence of *CYP2C19* genotype, with gIMs showing higher dose-normalized venlafaxine/ODV MR compared to gRMs, suggests a secondary but clinically relevant role of CYP2C19 in venlafaxine biotransformation [22,23]. Supporting this, data from a TDM service involving 1000 Norwegian patients reported that individuals carrying combined genotypes of *CYP2D6* IMs and *CYP2C19* PMs, as well as those who were PMs for both enzymes, exhibited approximately 4-fold and 13-fold higher dose-adjusted venlafaxine serum concentrations, respectively, compared with individuals classified as NMs for both enzymes. *CYP2D6*, *CYP2C19,* and combined *CYP2D6 + CYP2C19* genotypes accounted for approximately 24%, 11%, and 46% of the interindividual variability in dose-adjusted venlafaxine serum concentrations, respectively, underscoring the importance of genotyping both the drug-metabolizing enzymes for accurate pharmacokinetic predictions [22]. Future studies with larger cohorts should further evaluate the combined impact of *CYP2D6* and *CYP2C19* gPMs on venlafaxine/ODV MR. Evidence from four double-blind, placebo-controlled trials in patients with major depressive disorder suggested that *CYP2D6* NMs showed greater therapeutic response compared to PMs, highlighting that *CYP2D6* phenotype may predict venlafaxine efficacy [40]. The frequencies of *CYP2D6* and *CYP2C19* PM genotypes observed in this study cohort closely align with those reported in the European population [41,42,43,44]. In contrast, *CYP2C9* genotypes showed no significant effect on the dose-normalized venlafaxine/ODV MR, confirming its minor role in venlafaxine biotransformation [7]. There were no statistically significant changes in dose-normalized venlafaxine/ODV MR at an individual *CYP2D6*, *CYP2C19*, or *CYP2C9* SNP level. These findings highlight the clinical utility of incorporating *CYP2D6* and *CYP2C19* genotyping for personalized venlafaxine dosing. Patients with PM genotype may require lower starting doses or earlier TDM to avoid exceeding the laboratory alert threshold and prevent venlafaxine dose-related ADRs. The venlafaxine/ODV MR was selected as the primary pharmacokinetic endpoint because it reliably reflects the CYP2D6 enzyme’s activity and/or genotype [29,45] and can be derived from single steady-state trough concentrations under routine clinical conditions. While classical pharmacokinetic parameters such as maximum plasma concentration (C_max_) and area under the curve (AUC) provide comprehensive exposure estimates, their assessment usually requires relatively more intensive sampling. Moreover, the dose-normalized venlafaxine/ODV MR may serve as a surrogate marker of *CYP2D6* activity in settings where genotyping is not readily available. The dose-normalized venlafaxine/ODV MR minimizes variability related to venlafaxine dose and sampling time, thereby improving interpretability when evaluating genotype–phenotype relationships in naturalistic clinical settings. Integrating these combined plasma concentration monitoring and PGx strategies into implementation programs, such as MedeA in Spain [46], could support the clinical adoption of PGx and enable more guided and effective prescribing. Systematic evaluation of MR can be used to monitor potential interactions when another drug that is metabolized by the same enzyme is co-administered, a scenario common in psychiatric patients.

Several clinical and demographic factors have also been reported to influence the variability in venlafaxine pharmacokinetics [47,48]. Data from a Danish TDM service (n = 1077) reported that dose-normalized plasma concentrations of venlafaxine and ODV were markedly higher in patients aged >64 years [49]. Age-related increases in venlafaxine exposure were particularly pronounced in *CYP2D6* gPMs, with up to an eightfold elevation observed in elderly patients (>65 years), compared with those <40 years [50]. Drug–drug interactions have been identified as a risk factor for fatal venlafaxine toxicity [51]. Several co-medications have the potential to interact with venlafaxine through pharmacokinetic or pharmacodynamic drug–drug interaction mechanisms [52]. Concomitant medications such as valproic acid and clozapine have been reported to affect dose-corrected serum concentrations of ODV and the ODV/venlafaxine MR [53]. Furthermore, a recently published risk prediction model reported that an average daily venlafaxine dose ≥225 mg, renal impairment, and concomitant use of CYP2D6 inhibitors were independent predictors for plasma concentrations exceeding the laboratory alert threshold [54]. A recent prediction model using data from 330 inpatients diagnosed with depression by machine learning and deep learning techniques identified venlafaxine daily dose, sex, age, hyperlipidemia, and adenosine deaminase as potential variables that could affect venlafaxine active moiety concentration. However, this study tested only 12 demographic and clinical variables and did not include PGx data [55]. Among clinical variables, we found only sex to be associated with a statistically significant dose-normalized venlafaxine/ODV MR. However, this finding should be interpreted cautiously, given the small number of male patients. The observed sex-related differences highlight the need for further large, well-controlled studies to determine whether sex related metabolic differences warrant dose modifications or tailored monitoring strategies in clinical practice. Other demographic and clinical factors, such as age, BMI, smoking status, comorbidity burden, diabetes mellitus, hypertension, or polypharmacy/hyper-polypharmacy, did not have any significant influence on the dose-normalized venlafaxine/ODV MR in this cohort. Patients treated with venlafaxine alone showed better antidepressant response, greater remission of symptoms, fewer adverse events, and lower healthcare costs, compared to those who were concomitantly administered CYP2D6 substrates or inhibitors [56]. Weak CYP2D6 and CYP2C9, as well as weak to moderate CYP2C19 inhibitor use, did not increase dose-normalized venlafaxine/ODV MRs in this cohort.

Although this study was conducted in routine clinical settings, its main limitation was a small sample size, which may have reduced statistical power to detect significant associations between selected variables and the dose-normalized venlafaxine/ODV MR. Due to the small sample size, particularly within certain CYP genotype-predicted metabolizer subgroups, the study findings should be interpreted as exploratory and hypothesis-generating, rather than confirmatory evidence for clinical decision-making. Replication in a larger and ethnically diverse population is warranted to confirm the generalizability of these results. Further large-sample-size studies are needed to evaluate the impact of drug–drug interaction-mediated phenoconversion in patients receiving strong CYP inhibitors. None of the patients in the study were on concomitant medications that were strong inhibitors of CYP2D6, CYP2C19, or CYP2C9 enzymes. Additionally, the association between PGx or pharmacokinetics with ADR or treatment outcomes was not explored in this study. Despite these limitations, this study provides important evidence on the potential role of *CYP2D6* and *CYP2C19* genotypes on venlafaxine metabolism. Current regulatory and PGx guidelines specify *CYP2D6* as the primary PGx biomarker for venlafaxine [25,26,27,28], with the present study providing supportive mechanistic evidence for this. Although the study findings do not support immediate regulatory label changes or dosage recommendations, given the exploratory nature and smaller sample size, they suggest that *CYP2C19* genotype may be considered a secondary biomarker in future regulatory or guideline updates, subject to validation from large, outcome-linked studies. In antidepressant therapy, particularly with venlafaxine, the primary clinical challenge is not the lack of formal PGx-based dosing recommendations, but the substantial interindividual variability in exposure and treatment response observed in real-world settings. By integrating PGx, TDM, and clinical covariates, our study provides actionable insights that can support earlier identification of patients at risk of suboptimal exposure, adverse effects, or treatment failure, even within the dosing ranges currently recommended by regulatory agencies. This type of evidence is essential for improving clinical decision making, optimizing treatment selection, and guiding personalized follow-up strategies, areas where regulatory labels are not designed to provide granular guidance. Thus, the value of this study lies in enhancing clinical interpretability and implementation feasibility, rather than prompting immediate label changes. Future research should incorporate phenoconversion-aware algorithms integrating PGx and TDM, supported by validation in larger and more diverse populations to optimize venlafaxine dosing.

## 4. Materials and Methods

### 4.1. Research Protocol, Patient Population, and Ethical Committee Approval

This prospective study included patients recruited through the MedeA PGx Implementation Strategy (Extremadura, Spain) [46] between September 2023 and January 2025, following the same clinical workflow previously described [37]. Patients were eligible for inclusion if they were residents of Extremadura, attended the Extremadura Health Service (SES), were prescribed venlafaxine, and adhered to the prescribed treatment regimen. Pregnant women were excluded from the study. All patients provided written informed consent prior to their inclusion in the study. Clinical and demographic data (including age, sex, BMI, smoking status, comorbidities, polypharmacy (≥5 medications), hyperpolypharmacy (≥10 medications), and CYP inhibitor exposure were collected during clinical assessment and extracted from electronic medical records. The study adhered to the principles of the Declaration of Helsinki and received approval from the Ethics Committee for investigation with Medicines of Cáceres (CEIm) [No. 052-2021].

### 4.2. Genotyping and Phenotyping Analysis

Genotyping of *CYP2D6*, *CYP2C19*, and *CYP2C9* (Supplementary Table S1: Supplementary File S1) was performed using commercially available Taqman^®^ assays (Applied Biosystems, Foster City, CA, USA) and the following thermocycling conditions were applied: 10 min for initial denaturation at 95 °C, followed by 40 denaturation cycles of 15 s at 92 °C and annealing at 60 °C for 1 min. Allele discrimination was performed for 30 s at 60 °C. All assays included negative (no DNA) and positive (heterozygous and/or homozygous) control samples. Genotypes were assigned based on “key” single-nucleotide variants defining the alleles of interest (Supplementary Table S1: Supplementary File S1). Analysis of copy number variations (*CYP2D6* gene duplications and CYP2D6*5 deletions) was performed using a previously described methodology [57]. Metabolic phenotypes were classified from genotyping according to current guidelines [58], *CYP2D6* gPM (AS = 0), gIM (AS = 0.25–1), gNM (AS = 1.25–2.25), and gUM (AS > 2.25). *CYP2C19* phenotypes were classified as: gPMs (two no-function alleles), gIMs (one no-function allele plus one function allele), gNMs (two normal function alleles), rapid metabolizers (gRMs) (one normal function allele plus one increased function allele), and gUMs (two increased function alleles) [59]. *CYP2C9* phenotypes were predicted as follows: gPMs (AS = 0 or 0.5), gIMs (AS = 1 or 1.5), and gNMs (AS = 2) [60]. Concomitant drug use was not considered in the determination of MR.

### 4.3. Pharmacokinetic Profiling

Blood samples were collected in the morning under steady-state conditions, immediately before administration of the subsequent dose. Plasma samples were centrifuged at 3500 rpm for 13 min and stored at −20 °C until they were analyzed. A validated high-performance liquid chromatography (HPLC) commercial kit was used for venlafaxine and desvenlafaxine estimation (Chromsystems Instruments & Chemicals GmbH, Munich, Germany) [61].

Plasma samples, calibration standards, and quality control samples were processed using a protein precipitation procedure. Briefly, 50 µL of plasma was placed into a 1.5 mL microcentrifuge tube, followed by the addition of 25 µL of extraction buffer (Ref. 92005). After a 2 min incubation period, 250 µL of the internal standard solution was added, and the samples were mixed by vortexing for 30 s. The mixture was centrifuged at 13,000 rpm for 7 min, and finally, 150 µL of the resulting supernatant was transferred into an opaque glass vial and mixed with 150 µL of dilution buffer 1 (Ref. 92007). Chromatographic separation was conducted using an Agilent 1200 Series HPLC system (Agilent, Santa Clara, CA, USA) equipped with a binary pump, autosampler, degasser, and column oven. Analytes were separated on a TDM MasterColumns A (50 mm × 2 mm internal diameter; 3 μm) from Chromsystems MassTox^®^ (Ref. 92110) that was kept at a constant temperature of 30 °C. Detection was performed using an API2000 triple quadrupole mass spectrometer from AB Sciex (Framingham, MA, USA) equipped with an atmospheric pressure electrospray ionization interface was used for the mass analysis and detection, and operated with Analyst software (version 1.5.1).

### 4.4. Statistical Analysis

The Shapiro–Wilk test was applied to assess data normality. Associations between total daily venlafaxine dose and plasma concentrations were evaluated using Spearman’s correlation. The dose-normalized venlafaxine/ODV MR was compared across *CYP2D6*, *CYP2C19*, and *CYP2C9* genotype groups using the Kruskal–Wallis test followed by Dunn’s multiple comparisons test. A comparison of dose-normalized venlafaxine/ODV MR between *CYP2D6* poor and non-poor metabolizers was performed using the Mann–Whitney test. Group comparisons for demographic and clinical variables were also conducted using the Mann–Whitney test. Statistical significance was defined as *p* < 0.05. All statistical analyses were performed using GraphPad Prism (version 10.6.1) software.

## 5. Conclusions

In the present study, *CYP2D6* and *CYP2C19* PGxs were identified as key determinants of the dose-normalized venlafaxine/ODV MR, underscoring their central roles in venlafaxine metabolism. The study’s findings provide preliminary evidence on the potential utility of PGx and plasma concentration monitoring to inform personalized venlafaxine dosing in clinical practice. Future research should be conducted in larger and more diverse populations and should incorporate drug–drug interaction-mediated phenoconversion-aware algorithms to optimize venlafaxine dosing.

## Figures and Tables

**Table 1:** Influence of demographic and clinical factors on the dose-normalized venlafaxine/ODV MR.

Variables	Subcategories	N	Median (Q1, Q3) of Dose-Normalized Venlafaxine/ODV MR	Significance (*p*-Value)
Age (years)	≥65	11	0.0006 (0.0005, 0.0008)	0.1881
	<65	18	0.0012 (0.0005, 0.0054)	
Sex	Male	4	0.0115 (0.0054, 0.0504)	0.0006 *
	Female	25	0.0006 (0.0005, 0.0015)	
Body mass index (kg/m^2^)	<25	7	0.0008 (0.0005, 0.0016)	0.6005
	≥25	22	0.0007 (0.0004, 0.00251)	
Smokers *	Smokers	8	0.0011 (0.0005, 0.0075)	0.4688
	Non-smoker/Ex-smokers	20	0.0007 (0.0005, 0.0017)	
	No	22	0.0008 (0.0005, 0.0024)	
Comorbidities	<5	14	0.0012 (0.0004, 0.0054)	0.2172
	≥5	15	0.0006 (0.0005, 0.0015)	
Diabetes mellitus	Yes	8	0.0011 (0.0005, 0.0018)	0.8672
	No	21	0.0006 (0.0005, 0.0030)	
Hypertension	Yes	14	0.0007 (0.0004, 0.0025)	0.9145
	No	15	0.0006 (0.0005, 0.0018)	

* Smoking status was unknown for 1 patient.

**Table 2:** Dose-normalized venlafaxine/ODV MR stratified by polypharmacy status and CYP inhibitor exposure.

Variable	Subcategories	N	Median (Q1, Q3) of Dose-Normalized Venlafaxine/ODV MR
Polypharmacy	Yes	10	0.0006 (0.0002, 0.0025)
	No	19	0.0008 (0.0005, 0.0018)
Hyperpolypharmacy	Yes	7	0.0006 (0.0003, 0.0019)
	No	22	0.0008 (0.0005, 0.0024)
CYP2D6 inhibitor	Weak inhibitor	1	0.0001 [N/A]
	No inhibitors	28	0.0007 (0.0005, 0.0019)
CYP2C19 inhibitor	Weak/moderate	10	0.0007 (0.0005, 0.0071)
	No inhibitors	19	0.0008 (0.0005, 0.0016)
CYP2C9 inhibitor	Weak inhibitor	2	0.0007 (0.0006, 0.0008)
	No inhibitor	27	0.0007 (0.0005, 0.0019)

[N/A]: Not Applicable.

## Data Availability

The original contributions presented in this study are included in the article/Supplementary Material. Further inquiries can be directed to the corresponding authors.

## Supplementary Materials

The following supporting information can be downloaded at https://www.mdpi.com/article/10.3390/ph19020209/s1, Table S1: *CYP2D6*, *CYP2C19*, and *CYP2C9* variants and their corresponding Taqman^®^ gene expression assays utilized for real-time PCR genotyping; Table S2: *CYP2D6* allele frequencies; Table S3: *CYP2D6* genotype frequencies; Table S4: *CYP2C9* allele frequencies; Table S5: *CYP2C9* genotype frequencies; Table S6: *CYP2C19* allele frequencies; Table S7: *CYP2C19* genotype frequencies; Table S8: Dose-normalized venlafaxine/ODV MR based on *CYP2D6*, *CYP2C9*, and *CYP2C19* SNPs; Table S9: Dose-normalized venlafaxine/ODV MR based on total venlafaxine daily dose.

## Abbreviations

The following abbreviations are used in this manuscript:

SNR1	Serotonin–norepinephrine reuptake inhibitor
SSRI	Selective serotonin reuptake inhibitors
ODV	O-desmethylvenlafaxine
ADRs	Adverse drug reactions
TDM	Therapeutic drug monitoring
PGx	Pharmacogenetic
AS	Activity scores
MR	Metabolic ratio
gPM	Poor metabolizers
BMI	Body mass index
gIM	Intermediate metabolizer
gNM	Normal metabolizer
gRM	Rapid metabolizer
gUM	Ultrarapid metabolizer
SNP	Single nucleotide polymorphism
AGNP	Arbeitsgemeinschaft für Neuropsychopharmakologie und Pharmakopsychiatrie
HPLC	High-performance liquid chromatography
