# Supplementary material for: Influence of CYP2D6, CYP2C19, and CYP2C9 Pharmacogenetics and Clinical Factors on Dose-Normalized Venlafaxine/O-Desmethylvenlafaxine Metabolic Ratio in Spanish Patients
Source: Pharmaceuticals (Basel). 2026 Jan 26;19(2):209. doi: 10.3390/ph19020209 (PMC12944365; doi:10.3390/ph19020209)
Supplement: Supplementary file 1 [file pharmaceuticals-19-00209-s001.zip › pharmaceuticals-4100501-supplementary.pdf]

Supplementary File S1

Table S1. *CYP2D6, CYP2C19*, and *CYP2C9* variants and their corresponding Taqman® assays utilized for Real-Time PCR genotyping

CYP gene	CYP alleles	rs ID	Nucleotide change	Allele functional status	Activity score	Taqman® assay ID
CYP2D6	**2*	rs16947	2851C>T	Normal	1	C__27102425_10
		rs1135840	4181G>C		1	C__27102414_10
	**3*	rs35742686	2550delA	None	0	C__32407232_50
	**4*	rs3892097	1847G>A	None	0	C__27102431_D0
	**6*	rs5030655	1707T>del	None	0	C__32407243_20
	**9*	rs5030656	2616delAAG	Decreased	0.25	C__32407229_60
	**10*	rs1065852	100C>T	Decreased	0.25	C__11484460_40
	**17*	rs28371706	1022C>T	Decreased	0.5	C___2222771_A0
	**35*	rs769258	31G>A	Normal	1	C_27102444_F0
	**41*	rs28371725	2989G>A	Decreased	0.25	C__34816116_20
CYP2C9	**2*	rs1799853	3608C>T	Decreased	0.5	C_25625805_10
	**3*	rs1057910	42614A>C	None	0	C_27104892_10
	**5*	rs28371686	42619C>G	Decreased	0.5	C_27859817_40
	**6*	rs9332131	10601delA	None	0	C__32287221_20
	**8*	rs7900194	3627G>A	Decreased	0.5	C__25625804_10
CYP2C19	**2*	rs4244285	19154G>A	None		C__25986767_70
	**3*	rs4986893	17948G>A	None		C__27861809_10
	**4*	rs28399504	1A>G	None		C__30634136_10
	**5*	rs56337013	90033C>T	None		C__27861810_10
	**17*	rs12248560	-806C>T	Increased		C____469857_10

Table S2. *CYP2D6* allele frequencies

***CYP2D6* alleles**	**Frequency**
wt	0.3448
*2	0.1896
*4	0.1551
*4J	0.0172
*5	0.0344
*9	0.0344
*10	0.0344
*35	0.0689
*41	0.1034
*2x2	0.0172

Table S3. Frequencies of *CYP2D6* genotypes

***CYP2D6* genotypes**	**Frequency**
*2/*2	0.0344
*2/*35	0.0689
*2/*4	0.0689
*2/*5	0. 0344
*2x2/*10	0.0344
*4/*35	0.0344
*4/*4	0.0344
*4/*5	0.0344
*41/*41	0.0344
*4J/*10	0.0344
*9/*41	0.0344
wt/*2	0.1379
wt/*35	0.0344
wt/*4	0.1034
wt/*41	0.1034
wt/*9	0.0344
wt/wt	0.1379

Table S4. *CYP2C9* allele frequencies

***CYP2C9* allele**	**Frequency**
*2	0.1724
*3	0.0689
wt	0.7586

Table S5. *CYP2C9* genotype frequencies

***CYP2C9* genotype**	**Frequency**
*2/*3	0.0689
wt/*2	0.2758
wt/*3	0.0689
wt/wt	0.5862

Table S6. *CYP2C19* allele frequencies

***CYP2C19* allele**	**Frequency**
wt	0.6724
*2	0.1206
*4	0.0172
*17	0.1896

Table S7. *CYP2C19* genotypes frequencies

***CYP2C19* genotype**	**Frequency**
*17/*17	0.0344
*2/*17	0.0344
*2/*2	0.0344
wt/*4	0.0344
wt/*17	0.2758
wt/*2	0.1379
wt/wt	0.4482

Table S8. Dose-normalized venlafaxine/ODV MR based on *CYP2D6*, *CYP2C9*, and *CYP2C19* SNPs

Sl. No.	Gene	SNP	SNP status	N	Median (Q1, Q3) of dose-normalized venlafaxine/ODV MR	Significance (*p*-value)
1	*CYP2D6*	rs16947	WT	11	0.0006 (0.0005, 0.0019)	0.8771
			HT/HOM	18	0.0007 (0.0005, 0.0022)	
2		rs1135840	WT	5	0.0006 (0.0003, 0.0321)	0.8889
			HT/HOM	24	0.0007 (0.0005, 0.0018)	
3		rs35742686	WT	29	0.0007 (0.0005, 0.0018)	-
			HT/HOM	0	-	
4		rs3892097	WT	20	0.0007 (0.0005, 0.0017)	0.8714
			HT/HOM	9	0.0006 (0.0004, 0.0030)	
5		rs5030655	WT	29	0.0007 (0.0005, 0.0018)	-
			HT/HOM	0	-	
6		rs5030656	WT	27	0.0006 (0.0005, 0.0016)	0.1773
			HT/HOM	2	0.0031 (0.0018, 0.0044)	
7		rs1065852	WT	19	0.0008 (0.0005, 0.0018)	0.8747
			HT/HOM	10	0.0007 (0.0004, 0.0025)	
8		rs28371706	WT	29	0.0007 (0.0005, 0.0018)	-
			HT/HOM	0	-	
9		rs769258	WT	25	0.0006 (0.0005, 0.0030)	0.8311
			HT/HOM	4	0.0008 (0.0003, 0.0013)	
10		rs28371725	WT	24	0.0007 (0.0005, 0.0016)	0.4823
			HT/HOM	5	0.0044 (0.0003, 0.0120)	
11	*CYP2C9*	rs1799853	WT	19	0.0008 (0.0005, 0.0018)	>0.9999
			HT/HOM	10	0.0006 (0.0004, 0.0068)	
12		rs1057910	WT	25	0.0007 (0.0005, 0.0017)	0.6040
			HT/HOM	4	0.0079 (0.0003, 0.0507)	
13		rs28371686	WT	29	0.0007 (0.0005, 0.0018)	-
			HT/HOM	0	-	
14		rs9332131	WT	29	0.0007 (0.0005, 0.0018)	-
			HT/HOM	0	-	
15		rs7900194	WT	29	0.0007 (0.0005, 0.0018)	-
			HT/HOM	0	-	
16	*CYP2C19*	rs4244285	WT	23	0.0006 (0.0005, 0.0019)	0.4774
			HT/HOM	6	0.0008 (0.0006, 0.0033)	
17		rs4986893	WT	29	0.0007 (0.0005, 0.0018)	-
			HT/HOM	0	-	
18		rs28399504	WT	28	0.0007 (0.0005, 0.0019)	-
			HT/HOM	1	0.0003 [N/A]	
19		rs56337013	WT	29	0.0007 (0.0005, 0.0018)	-
			HT/HOM	0	-	
20		rs12248560	WT	18	0.0006 (0.0004, 0.0024)	0.8079
			HT/HOM	11	0.0008 (0.0005, 0.0019)	

Abbreviations: WT: Wildtype, HT: Heterozygous, HOM: Homozygous mutant

Table S9. Dose-normalized venlafaxine/ODV MR based on total venlafaxine daily dose

Sl. No.	Total venlafaxine dose (mg)	N	Median (Q1, Q3) of dose-normalized venlafaxine/ODV MR
1	37.5 mg	1	0.0001 (N/A)
2	75 mg	5	0.0007 (0.0006, 0.0029)
3	150 mg	13	0.0009 (0.0005, 0.0093)
4	225 mg	7	0.0006 (0.0005, 0.0018)
5	300 mg	3	0.0006 (0.0001, 0.0086)
